# Lipocalin 2 is protective against *E. coli *pneumonia

**DOI:** 10.1186/1465-9921-11-96

**Published:** 2010-07-15

**Authors:** Hong Wu, Eric Santoni-Rugiu, Elisabeth Ralfkiaer, Bo T Porse, Claus Moser, Niels Høiby, Niels Borregaard, Jack B Cowland

**Affiliations:** 1 Department of Clinical Microbiology, Rigshospitalet, Copenhagen, Denmark; 2 Department of Pathology, Rigshospitalet, Copenhagen, Denmark; 3 Section for Gene Therapy Research and Biotech Research and Innovation Centre (BRIC), University of Copenhagen, Copenhagen, Denmark; 4 Granulocyte Research Laboratory, Rigshospitalet, Copenhagen, Denmark

## Abstract

**Background:**

Lipocalin 2 is a bacteriostatic protein that binds the siderophore enterobactin, an iron-chelating molecule produced by *Escherichia coli *(*E. coli*) that is required for bacterial growth. Infection of the lungs by *E. coli *is rare despite a frequent exposure to this commensal bacterium. Lipocalin 2 is an effector molecule of the innate immune system and could therefore play a role in hindering growth of *E. coli *in the lungs.

**Methods:**

Lipocalin 2 knock-out and wild type mice were infected with two strains of *E. coli*. The lungs were removed 48 hours post-infection and examined for lipocalin 2 and MMP9 (a myeloid marker protein) by immunohistochemical staining and western blotting. Bacterial numbers were assessed in the lungs of the mice at 2 and 5 days after infection and mortality of the mice was monitored over a five-day period. The effect of administering ferrichrome (an iron source that cannot be bound by lipocalin 2) along with E.coli was also examined.

**Results:**

Intratracheal installation of *E. coli *in mice resulted in strong induction of lipocalin 2 expression in bronchial epithelium and alveolar type II pneumocytes. Migration of myeloid cells to the site of infection also contributed to an increased lipocalin 2 level in the lungs. Significant higher bacterial numbers were observed in the lungs of lipocalin 2 knock-out mice on days 2 and 5 after infection with *E. coli *(p < 0.05). In addition, a higher number of *E. coli *was found in the spleen of surviving lipocalin 2 knock-out mice on day 5 post-infection than in the corresponding wild-type mice (p < 0.05). The protective effect against *E. coli *infection in wild type mice could be counteracted by the siderophore ferrichrome, indicating that the protective effect of lipocalin 2 depends on its ability to sequester iron.

**Conclusions:**

Lipocalin 2 is important for protection of airways against infection by *E. coli*.

## Background

Despite frequent exposure of the body to commensal bacteria from the intestinal system, such as *E.coli*, extraintestinal infections are quite rare. The lungs are continuously exposed to bacteria including *E.coli *and must therefore be able to prevent bacterial growth. The innate immune system has evolved in higher eukaryotes as the first line of defence against potential microbial pathogens. The cells of the epithelial lining are important players in this scenario as they produce many antimicrobial proteins in response to the invading microorganisms. Microorganisms are recognized by pathogen-associated molecular patterns (PAMPs) that specifically expressed on bacteria and fungi [1]. These PAMPs are recognized by pathogen recognizing receptors (PRRs) on epithelial cells and/or interstitial macrophages and dendritic cells [1]. In the former case, an intracellular signal is generated that leads to a direct response by the epithelial cells. In the latter case, ligation of PAMPs to receptors on leukocytes stimulates synthesis of pro-inflammatory cytokines that in turn will induce a response in epithelial cells [1,2]. In both instances this will lead to *de novo *synthesis and secretion of antimicrobial proteins to the immediate surroundings of the epithelium where these proteins will exert their biological functions. Specialized mobile phagocytes, such as neutrophils and monocytes, will appear at the site of infection to combat the pathogens, not least by exocytosing microbicidal proteins from their stores in intracellular granules. Antimicrobial proteins similar to those stored in phagocytes are induced in epithelial cells by contact with microorganisms or by cytokines. [3].

One such antimicrobial protein is lipocalin 2. Lipocalin 2 is a 25 kDa glycoprotein first identified as a matrix protein of specific granules of human neutrophils [4] and therefore originally named neutrophil gelatinase-associated lipocalin (NGAL) [4]. It was later found that lipocalin 2 is also strongly upregulated in epithelial cells during inflammation [2,5-8]. Lipocalin 2 belongs to the lipocalin superfamily whose members share a barrel-shaped tertiary structure with a hydrophobic pocket that can bind lipophilic molecules [9]. The ligand of lipocalin 2 is bacterial ferric siderophores. Siderophores are generated by microorganisms when lack of soluble iron becomes a limiting factor for their growth. Siderophores are the strongest iron chelators known and are used by bacteria for uptake of iron [10,11]. Binding of siderophores by lipocalin 2 deprives bacteria of iron and lipocalin 2 consequently acts as a bacteriostatic protein.

It has been demonstrated previously that lipocalin 2 is protective against infection by *E. coli *injected directly into the peritoneum [11]. That model, however, circumvents the important barrier against microbial infections provided by the epithelial lining of our mucous membranes. We therefore decided to investigate whether lipocalin 2 has a role in protection against *E. coli *when these are introduced in the airways and need to overcome the protection provided by the epithelial lining in order to establish infection. We demonstrate that intratracheal installation of *E. coli *induces strong expression of lipocalin 2 in the epithelial cells of the respiratory tract and that lack of lipocalin 2 expression results in increased morbidity and mortality of the infected mice. These data support the idea that the innate immune system is important for hindering infection by commensal bacteria such as E. coli.

## Methods

### Bacterial strains and culture conditions

The *E. coli *strains HB101 (ATCC 33694) and H9049 (a clinical isolate kindly provided by Dr. Alan Aderem, Institute for Systems Biology, Seattle, WA) were selected for the experiments, as they depend on enterobactin for uptake of iron. The bacteria were grown in Luria-Broth medium overnight with agitation at 37°C before being used for the experiments. The bacteria were harvested, resuspended in PBS, and the suspension of bacteria adjusted to the concentration required for the experiment. The titer of the bacteria was controlled by serial dilutions and cultures of the inoculum.

### Mouse model for lung infection

Eleven-week-old female lipocalin 2 (Lcn2) knock-out and wild-type littermates, both in a C57BL/6 background, were used for the experiments. The knock-out mice were kindly provided by Dr. Shizuo Akira, Osaka University, Osaka, Japan and Dr. Alan Aderem, Institute for Systems Biology, Seattle, WA. The Lcn2 knock-out mice used in the experiments had been back-crossed to C57BL/6 mice for 8 or 9 generations. A detailed protocol for bacterial inoculation has been described previously [12]. In brief, before surgical procedure, all mice were anesthetized by subcutaneous injection of a 1:1 mixture of etomidat (Janssen, Birkerød, Denmark) and midazolam (Roche, Hvidovre, Denmark) at a dose of 10 μl/g body weight. Tracheotomy was then performed and 40 μl bacterial suspension was instilled into the tracheal via a curved bead-tipped needle. The mice were infected with 4-8 × 10^7 ^*E. coli*/mouse. The incision was sutured with silk and healed without complications. The animals were sacrificed by 20% pentobarbital (DAK, Copenhagen, Denmark) at 2 μl/g body weight. Desferri-ferrichrome (without iron) and iron-loaded ferrichrome (both from EMC microcollections, Tübingen, Germany) were resuspended in sterile water and added to the bacterial suspension prior to infection of the mice. All animal experiments were conducted in accordance with the guidelines of the Danish Animal Ethics Committee.

### Lung and spleen bacteriologies

Quantification of bacteria in organs from challenged mice was performed as described previously [13]. In short, lungs or spleens were removed aseptically from the mice and immediately put into sterile containers with 5 ml of 4°C sterile PBS. Lung and spleen samples were homogenized with a blender (Heidolph, Struers, Denmark) at 4°C and series of diluted samples were plated on agar plates and incubated at 37°C for quantitative bacteriological examination after 20-24 hours of incubation. The resulting bacterial load is expressed as colony formation units (CFU)/lung or CFU/spleen.

### Immunhistochemical staining

Whole lungs and femur were removed from the mice and fixed overnight at 4°C in 10% buffered formalin. The bone tissue was decalcified by 4 mol/l formic acid and 0,5 mol/l natriumformate. Lung and bone tissues were then embedded in paraffin, and 4 μm-thick sections obtained by microtome were mounted onto coated glass slides and afterward deparaffinized and rehydrated according to standard protocols. Subsequently, the antigens of interest were retrieved in a microwave-oven in Tris/EGTA (TEG) buffer pH 9.0 for 18 min, at 600 watt. Then, the sections were let to cool-down in TEG buffer for 20 min, rinsed, and incubated in 3% H_2_O_2 _in methanol for 15 min to block endogenous peroxidase activity. For immunohistochemical detection of lipocalin 2, we used a rabbit polyclonal antibody (dilution 1:250) generated in our laboratory according to a protocol previously described [14]. Metalloproteinase-9 (MMP9) was detected using rabbit polyclonal anti-MMP9 antibody (Ab38898, Abcam, Biosite; dilution 1:2000) The antibodies were incubated in TBS with 1% BSA for 30 min. To rule out non-specific binding, rabbit serum collected before immunization with lipocalin 2 (pre-immune serum) and a nonspecific rabbit Ig (DAKO, no. X0903, Dako, Glostrup, Denmark) were used as negative controls for lipocalin 2 and MMP-9 antibodies, respectively, in the same dilutions as for the specific antibodies. DAKO Envision-System-horseradish peroxidase (HRP) (DakoCytomation, no. K4011) with diaminobenzidine as substrate chromogen was used according to manufacturer's instructions to visualize the binding of the primary antibodies. The samples were counterstained with Mayer's hematoxylin for 1 min.

### SDS-PAGE and immunoblotting

For immune-detection, the proteins from lung lysates were separated on a 4-12% NuPAGE Bis-Tris gel (Invitrogen) and electro-transferred to a Trans-Blot nitrocellulose membrane (Bio-Rad) according to the manufacturer's instructions. The membrane was blocked for 1 h with 5% skimmed milk and washed four times 5 min. in PBS with 0.5% BSA. The primary antibodies for lipocalin 2 (AF1857, R&D systems, dilution 1:1000), MMP9 (Ab38898, Abcam, dilution 1:1000), and β-Actin (13E5, Cell Signaling, dilution 1:5000)) were incubated overnight at 4°C in PBS with 0.5% BSA and then washed four times 5 min. in PBS with 0.5% BSA. The membranes were next incubated for 2 hours with the secondary antibody (peroxidase-conjugated goat anti-rabbit antibodies (P0448, DAKO, dilution 1:1000)), washed four times 5 min. in PBS with 0.5% BSA and visualized by chemiluminescence (SuperSignal West Pico, Thermo Scientific).

### Statistics

The unpaired differences in the continuous data between infected and non-infected mice were analyzed by the Mann-Whitney U-test. The software Statview (SAS Institute, Cary, NC) was used for the statistical analysis. Statistical significance was reported if *p *< 0.05 was achieved.

## Results

### Infection of the respiratory tract induces lipocalin 2 expression in bronchial epithelium and type II pneumocytes

**Figure 1:**
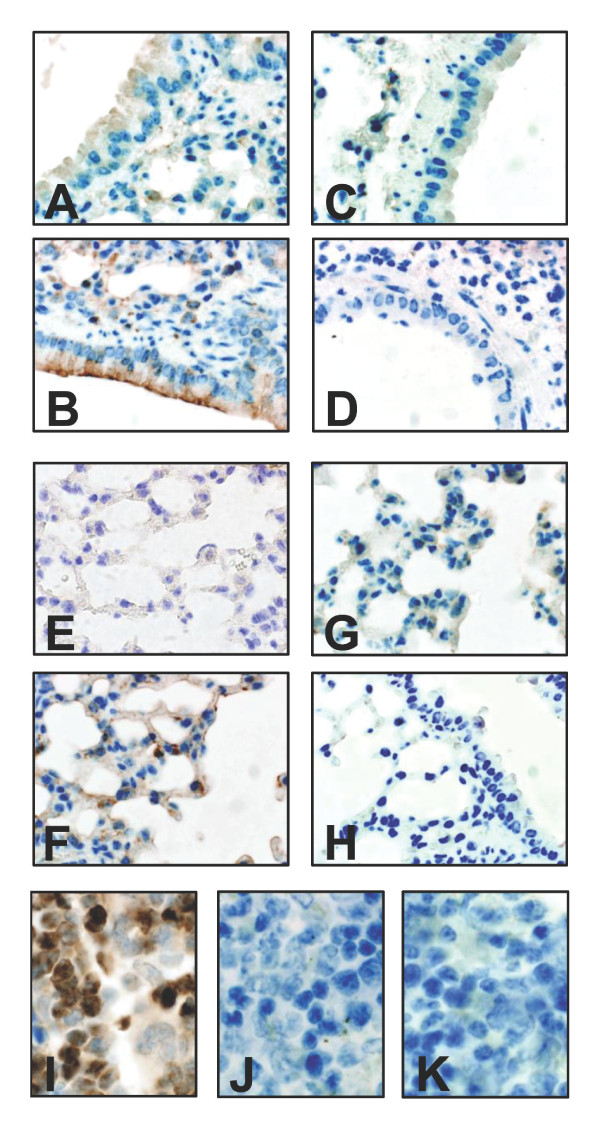
**Lipocalin 2 expression in the lungs of E. coli-infected mice**. Immunohistochemical staining using a polyclonal antibody against lipocalin 2 (diluted 1:250) on formalin-fixed lung sections removed 48 hours post-infection with *E. coli *H9049. Weak staining for lipocalin 2 is found in uninfected bronchial epithelium (A) and alveolear tissue (E) of wild-type C57BL/6 mice. Strong induction is seen following *E. coli *infection (4 × 10^7 ^CFU *E. coli *H9049/mouse) in wild-type mice (B and F) whereas no staining for lipocalin 2 is seen in infected Lcn2 knock-out mice (C and G). The specificity of the reaction is demonstrated by the lack of staining when using rabbit pre-immune serum (dilution 1:250) as negative control (D and H). Staining for lipocalin 2 was also observed in neutrophils in the bone marrow of wild-type mice (I) but not in Lcn2 knock-out mice (J) or in wild-type mice incubated with pre-immune serum (K).

We have previously demonstrated a strong up-regulation of lipocalin 2 in human bronchial epithelium in connection with bacterial infections [8]. To investigate whether this is the case also in a mouse model, we analysed lung sections of C57BL/6 mice that had been challenged by bacterial infection. As demonstrated by immunohistochemistry shown in fig. 1, a strong up-regulation of lipocalin 2 was observed at 48 hours post-infection in response to bacterial challenge with *E. coli *H9049. This was observed both in the bronchial epithelium (fig. 1B) and in type II pneumocytes of the alveoli as identified by their typical morphology (fig. 1F). In contrast, almost no staining for lipocalin 2 was observed in the bronchial epithelium (fig. 1A) or alveoli (fig. 1E) of uninfected wild-type mice. Besides the induced synthesis of lipocalin 2 in epithelial cells, lipocalin 2 is also expressed during neutrophil development in the bone marrow and stored in exocytosable granules [4]. Accordingly, positive staining for lipocalin 2 was seen in bone marrow neutrophils of wild-type mice (Fig. 1I). To examine the effect of lipocalin 2 in the defence against pulmonary bacterial infections, we employed a previously described lipocalin 2 knock-out mouse [11]. As expected, no staining for lipocalin 2 was observed in bronchial epithelium or lung alveoli of knock-out mice infected by bacteria, nor in bone marrow neutrophils (Fig. 1C, G, and 1J).

### The amount of lipocalin 2 in lung lysates increases dramatically following infection

**Figure 2:**
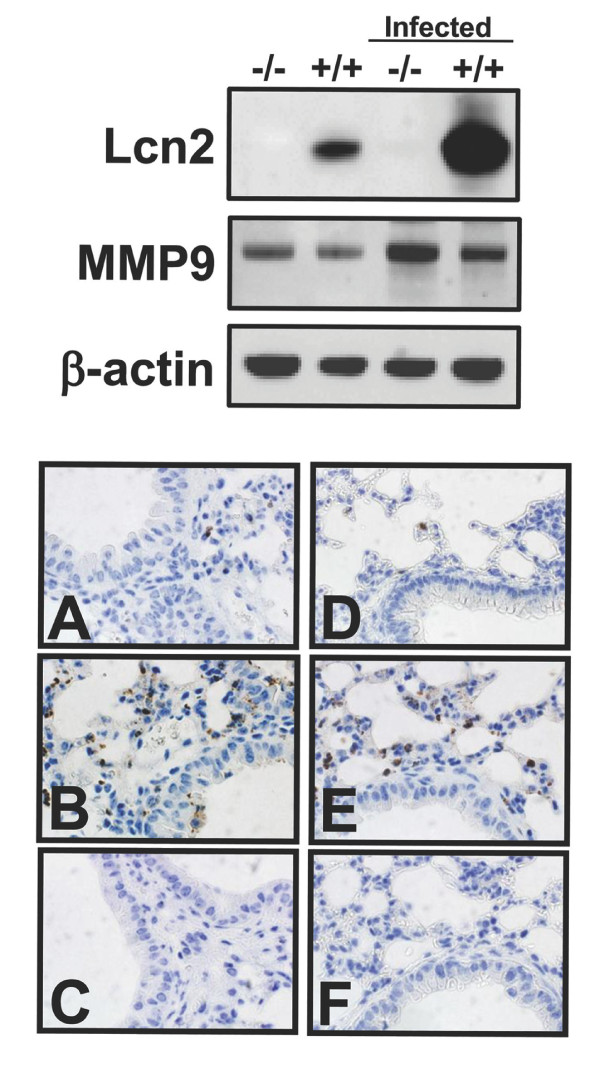
**MMP9 expression in the lungs of E. coli-infected mice**. *Top*. Western blot analysis for lipocalin 2 (Lcn2) and MMP9 (both antibodies diluted 1:1000) of whole lung lysates from uninfected and *E. coli*-infected (4 × 10^7 ^CFU *E. coli *H9049) wild-type (+/+) and Lcn2 knock-out (-/-) mice. Immunostaining for β-actin (dilution 1:5000) was included to assure equal loading of the samples. *Bottom*. Immunohistochemical staining for the neutrophil granule protein MMP9 (dilution 1:2000) on formalin-fixed lung sections of wild-type (A-C) and Lcn2 knock-out (D-F) mice. Only a few positive cells were found in the lungs of uninfected mice (A and D) whereas a larger number of cells were stained in the lungs of *E. coli *infected mice (4 × 10^7 ^CFU *E. coli *H9049/mouse) (B and E). No staining was seen when using a non-specific rabbit Ig as negative control (C and F).

In order to evaluate the level of lipocalin 2-induction following bacterial challenge of the lungs, we isolated protein from whole cell lysates of uninfected and infected lungs from both wild-type and lipocalin 2 knock-out mice. As seen in the immunoblot in figure 2, stronger expression of lipocalin 2 was observed in the infected wild-type mouse compared to the uninfected wild-type mouse. As expected, no expression of lipocalin 2 was observed in the knock-out mice, regardless of whether they were infected or not. Staining for the metalloproteinase 9 (MMP9), which is constitutively present in neutrophil granules, was performed to evaluate the influx of neutrophils into the tissues. An increase in staining was found for both infected wild-type and knock-out mice using MMP9 and cellular morphology as markers, indicating that migration of neutrophils to the infected lung was not abolished in the lipocalin 2 knock-out mouse (fig. 2B and 2E).

### Lipocalin 2 protects against lung infection by E. coli

**Figure 3:**
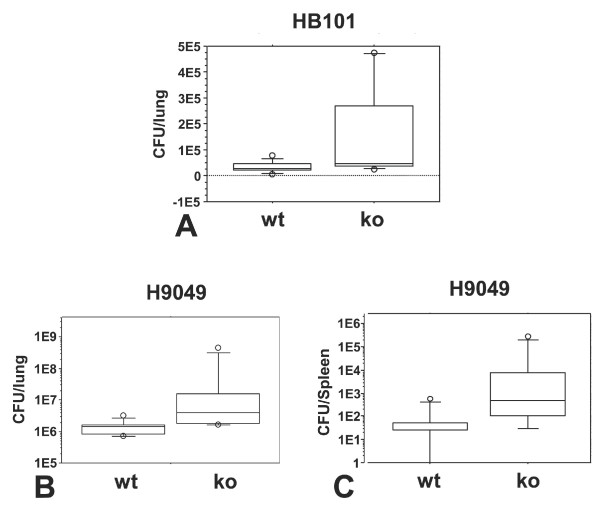
**Bacterial numbers in the lungs at 48 hours post-infection**. Data are presented as box plots showing CFU/lung (log 10 scale) in wild-type (wt) and Lcn2 knock-out (ko) mice infected with *E. coli *HB101 or H9049 (both 8 × 10^7 ^CFU/mouse) and tested after 48 hours. (A) For *E. coli *HB101, a statistical significant difference between the CFU in the lungs of wild-type (n = 9) and Lcn2 knock-out mice (n = 8) was observed (p < 0.05). No bacteria were measured in the spleen of these mice. For *E. coli *H9049, a significant difference was observed for CFU from the lungs (B) (p < 0.01) and spleen (C) (p < 0.05) of wild-type (n = 8) and Lcn2 knock-out (n = 8) mice.

We chose to test the susceptibility of the mice against two strains of *E. coli *that are dependent on enterobactin for iron uptake, namely HB101 [15] and H9049 [11]. We first examined the effect of a short-term infection (48 hours) of a lipocalin 2 knock-out mouse compared to wild-type littermates. At this time-point, no mice had succumbed to the infection, but a significant higher number of bacteria was found in the lungs of the knock-out mice (HB101, p = 0.048 and H9049, p = 0.0033) as seen in figure 3A and 3B. We also examined the spleen of these animals to determine whether the bacteria had been able to cross the epithelial lining of the lung and infect internal organs. No bacteria were found in the spleen of either type of mice inoculated with *E. coli *HB101 whereas infection of the spleen with a significant higher number of bacteria was observed in knock-out mice compared to the wild-type when these were challenged with *E. coli *H9049 (p = 0.019) (fig. 3C). These data clearly demonstrate that lipocalin 2 has a protective effect against this *E. coli *strains.

**Figure 4:**
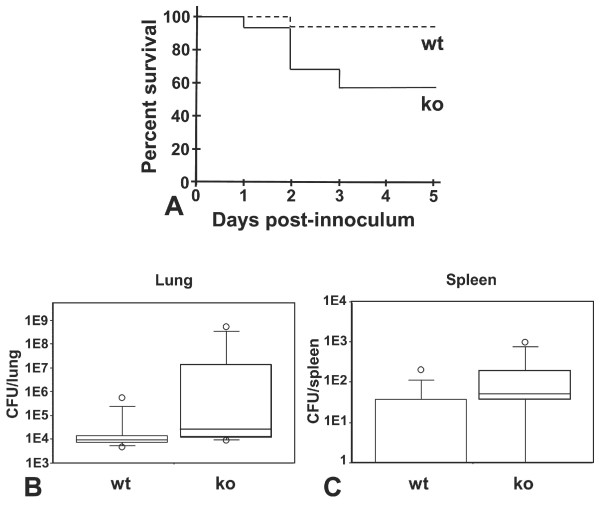
**Lcn2 knock-out mice are highly susceptible to lung infection with E. coli H9049**. (A) Survival curves of wild-type (wt) (n = 12) and lipocalin 2 knock-out (ko) (n = 16) mice infected with 4 × 10^7 ^CFU *E. coli *H9049/mouse demonstrating a significant higher mortality (p < 0.05) of the knock-out mice. Box plots showing bacterial numbers (CFUs) for the surviving mice (ko (n = 9) and wt (n = 11)) in the lungs (B) and spleens (C). A significantly higher number of bacteria were found in both the lungs (p < 0.05) and spleens (p < 0.05) of Lcn2 knock-out mice than in wild-type mice.

We investigated the effect of the *E. coli *infection after a longer incubation period. For this experiment, we decided to use *E. coli *H9049, as it appeared to be more virulent than HB101. After five days, almost half the knock-out mice (44%) had died in comparison to only one (8%) of the wild-type mice and the study was finalized (fig. 4A). We found a significantly higher bacterial load in the lungs of the surviving lipocalin 2 knock-out mice compared to the number of bacteria in the wild-type mice (p = 0.028) (fig. 4B). A higher bacterial load was also found in the spleen of knock-out mice compared to the wild-type (p = 0.024) (fig. 4C).

### The bacteriostatic effect of lipocalin 2 is dependent on its ability to bind siderophores

**Figure 5:**
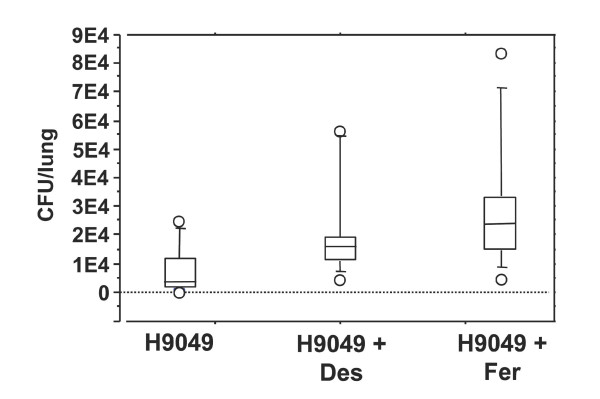
**The bacterial load increases in the lungs of mice administered ferrichrome**. Bacterial numbers (CFU) after 5 days in the lungs of wild-type mice infected with 4 × 10^7 ^CFU *E. coli *H9049/mouse alone (n = 9) or with 25 mmol desferri-ferrichrome (Des) (n = 10) or iron-loaded ferrichrome (Fer) (n = 13). Three of the mice infected with *E. coli *H9049 and iron-loaded ferrichrome died and bacterial loads were determined only for the 10 mice surviving to day 5. The number of bacteria in lungs of mice inoculated only with bacteria was significantly lower that in mice also receiving desferri-ferrichrome (p < 0.05) or iron-loaded ferrichrome (p < 0.01). The amount of CFU/lung of mice receiving desferri-ferrichrome + *E. coli *H9049 was significantly lower than the amount measured in the lungs of the surviving mice that had been administered *E. coli *H9049 + iron-loaded ferrichrome (p < 0.05). No bacteria were measured in the mice receiving only iron-loaded ferrichrome (n = 6).

It is known that lipocalin 2 is unable to bind all types of siderophores produced by microorganisms [16]. One example is ferrichrome, which is a siderophore of the hydroxymate type produced by fungi. Although *E. coli *does not produce ferrichrome it carries receptors for ferrichrome and is thus able to take up iron via this siderophore [17]. To demonstrate that the effect of lipocalin 2 is due to iron-depletion through binding of enterobactin, we infected wild-type C57BL/6 mice with *E. coli *H9049 with and without ferrichrome added to the bacterial innoculum. A significantly higher number of bacteria (p = 0.03) were observed five days after infection in the lungs of the mice that had received *E. coli *H9049 along with desferri-ferrichrome compared to the mice that only were exposed to the bacteria (fig. 5). This effect was even more pronounced if ferrichrome, pre-loaded with iron, was co-inoculated with the *E. coli *strain as 3 of the 13 mice died at days 2, 3, and 4, respectively. Furthermore, the bacterial load in the lungs of the surviving 10 mice was higher than in the mice only receiving *E. coli *H9049 (p = 0.01). A group of mice receiving ferrichrome, but no bacteria, was also included in the study. As expected, no bacteria were found in the lung lysates of these mice (data not shown).

## Discussion

The human orthologue of lipocalin 2, NGAL, is constitutively expressed in the goblet cells of trachea and is strongly upregulated in the epithelial lining of the upper airways and in type II pneumocytes of the alveoli following lung infection [8]. Expression of lipocalin 2 is induced in epithelial cells in an NF-κB dependent manner following stimulation with pro-inflammatory cytokines and constitutively secreted to the surroundings [2,8]. Furthermore, lipocalin 2 is stored in the specific granules of neutrophils from which it may be exocytosed when these cells have migrated to a site of infection [3,4].

Mice that do not express lipocalin 2 have previously been demonstrated to be more susceptibility towards intraperitoneal *E. coli *infections than wild type mice [11,18]. We show here that lipocalin 2 also plays a role in protection against *E. coli *when the bacteria are encountered on the epithelial surface of the lower airways. In the present study, the wild-type mice had a significantly lower pulmonary and spleen bacterial load in a pneumonia model with *E. coli *HB101 and H9049 at 48 hours compared to the Lcn2 knock-out mice. Furthermore, in the late pneumonic phase (5 days after intratracheal challenge with *E. coli *H9049), significant higher survival rates as well as a lower bacterial load in the lungs and spleens were found in wild-type mice compared to Lcn2 knock-out mice. These results indicate that lipocalin 2 has an important protective effect against lung infections caused by bacteria that produce siderophores, which are ligands for lipocalin 2. This is in accordance with the findings from other researchers [11,18].

Strong immunostaining for lipocalin 2 is seen in bronchial epithelial cells and in type II pneumocytes of the alveoli following infection with *E. coli*. A comparable increase in the amount of the neutrophil granule protein MMP9 was observed in the lung lysates of wild-type and Lcn2 knock-out mice by immunoblotting. This indicates that recruitment of myeloid cells to the infected lung was not impaired in the knock-out mice. This is supported by immunohistochemical staining of lung tissue from wild-type and Lcn2 knock-out mice where comparable levels of MMP9 positive cells are seen which were identified as neutrophils by morphology. Furthermore, the increase in lipocalin 2 expression between uninfected and *E. coli*-infected wild-type mice appeared to be more pronounced than the increase in MMP9 expression, which indicates that a considerable amount of the lipocalin 2 measured in these samples was secreted by the epithelial cells. A recent report describes induction of lipocalin 2 in lung cells following infection with *Klebsiella pneumonia *and points to a toll-like receptor 4 (TLR4)-mediated induction pathway [19]. Whether this mechanism also is involved in *E. coli*-induced lipocalin 2 expression is not known but as TLR4 is expressed both on epithelial cells and monocytes-derived dendritic cells of the airways [20], it is a plausible mechanism.

The data presented here suggest that the lipocalin 2 released locally in the lungs either by import of myeloid cells or generated by the epithelial cells is an important factor in preventing dissemination of an *E. coli *infection in mice and suggests that this may also be the case in humans. The bacteriostatic effect exerted by lipocalin 2 is caused by its ability to bind iron-loaded siderophores and thus sequester the iron needed for bacterial growth. Adding a siderophore that can be taken up by the bacteria but is unable to be bound by lipocalin 2 should therefore be able to counteract this effect. Ferrichrome fulfils these requirements as demonstrated in the intraperitoneal infection model [11]. This is also the case in the lung infection model presented here where a higher bacterial load was measured in the lungs of mice infected with a bacterial suspension containing ferrichrome than in the lungs of mice administered the same amount of bacteria without ferrichrome. The observation that an even higher bacterial load was observed in mouse lungs where iron-loaded ferrichrome was added to the bacteria rather than desferri-ferrichome further supports this notion.

Infection of the lungs with *Enterobacteriaceae *is much more common with *K. pneumonia *than with *E. coli*. A recent report demonstrated no difference between the degree of colonisation of *K. Pneumonia *in wild-type and lipocalin 2 knock-out mice [21]. This was due to the ability of this bacterium to form both a modified (glycosylated) form of enterobactin and a second siderophore, yersiniabactin (Ybt), of which neither can be bound by lipocalin 2. When testing a mutated form of *K. Pneumonia *that was unable to synthesize Ybt as well as glycosylate enterobactin, wildtype mice were able to combat infection with this bacterium whereas lipocalin 2 deficient mice were not [21]. This argues that it is the ability of this bacterium to use a modified enterobactin as well as a second type of siderophore as iron scavenger that make *K. Pneumonia *a pathogen of the lungs. The *E. coli *strain H9049 used in this study is also able to evade the bacteriostatic effect of lipocalin 2 if it acquires the *iroA *cluster that encodes the proteins involved in modification of enterobactin to the glycosylated form [22]. This was demonstrated in an intraperitoneal infection model where injection of *E. coli *H9049 carrying the iroA cluster caused a marked increase in the mortality of wild-type mice compared to mice receiving the unmodified form of H9049 [22]. It is thus possible for bacteria to evade the protective effect of lipocalin 2 either by biochemical modification of enterobactin or by acquiring iron by another method than chelation by enterobactin. This is likely to be a trait of many lung pathogens as exemplified by *Streptococcus pneumoniae *and *Haemophilus influenzae*. Both of these bacterial strains readily infect mice in an intranasal inoculation model despite a strong up-regulation of lipocalin 2 in the nasal epithelium in response to the infection [23]. The reason why these lung pathogens can evade the bacteriostatic effect of lipocalin 2 is that neither of these two bacterial strains produces siderophores nor use them for iron acquisition but instead have developed other means of iron uptake [23].

Infections of the respiratory system by *E. coli *do, however, occur, and may have severe implications in humans. *E. coli *lung infections or pneumonia are observed in patients with haematological diseases [24] and in patients that need mechanical ventilation in hospital ICU units [25]. Recently, a report was published describing a significantly higher number of *E. coli *or *Staphylococcus aureus *in microbiological samples from cases of sudden unexpected death in infancy (SUDI) than in infants whose death was due to a non-infectious cause [26]. It was suggested that the presence of *E. coli *could be associated with SUDI although a direct link was not demonstrated. It is known that the level of lipocalin 2 expression varies considerably between different individuals [27] and it may thus be possible that this could play a role in the susceptibility to *E. coli *infections. Our data demonstrate that the innate immune system plays a significant role in keeping infections by bacteria, which are normally considered to be non-pathogenic, at bay. If the innate immune system, on the other hand, is compromised then there is a risk that otherwise harmless commensal bacteria may cause infection of our body.

## Conclusion

Our data demonstrate that lipocalin 2 is important for hindering infection of the lungs by *E. coli*. *E. coli *is usually considered a non-pathogenic bacterium unless it has attained a specific trait that enables it to overcome the natural defence mechanisms of the body. Our data demonstrate that if the innate immune system is compromised - in this case by inactivating the gene encoding lipocalin 2 - then also normally non-pathogenic *E. coli *can become infectious. This underscores the importance of the innate immune system in the defence of the body against microorganisms

## Competing interests

The authors declare that they have no competing interests.

## Authors' contributions

HW has performed the experimental studies. ES-R and ER have performed the immuno-histochemical analysis. BTP has assisted with the mouse work. HW, CM, NH, NB, and JBC has designed the experimental set up, supervised the experimental work, and participated in preparation of the manuscript. All authors have read and approved the final manuscript.
